# Supplementary material for: Yiai Fuzheng decoction inhibits triple-negative breast cancer by remodeling the immune microenvironment
Source: Front Immunol. 2025 Sep 30;16:1615631. doi: 10.3389/fimmu.2025.1615631 (PMC12518410; doi:10.3389/fimmu.2025.1615631)
Supplement: Supplementary file 2 [file DataSheet1.zip › Supplementary File 2/Treatment/C_vs_D_vs_H/06_Pathway_Analysis/Enrichment_Analysis/predicted/Network.pdf]

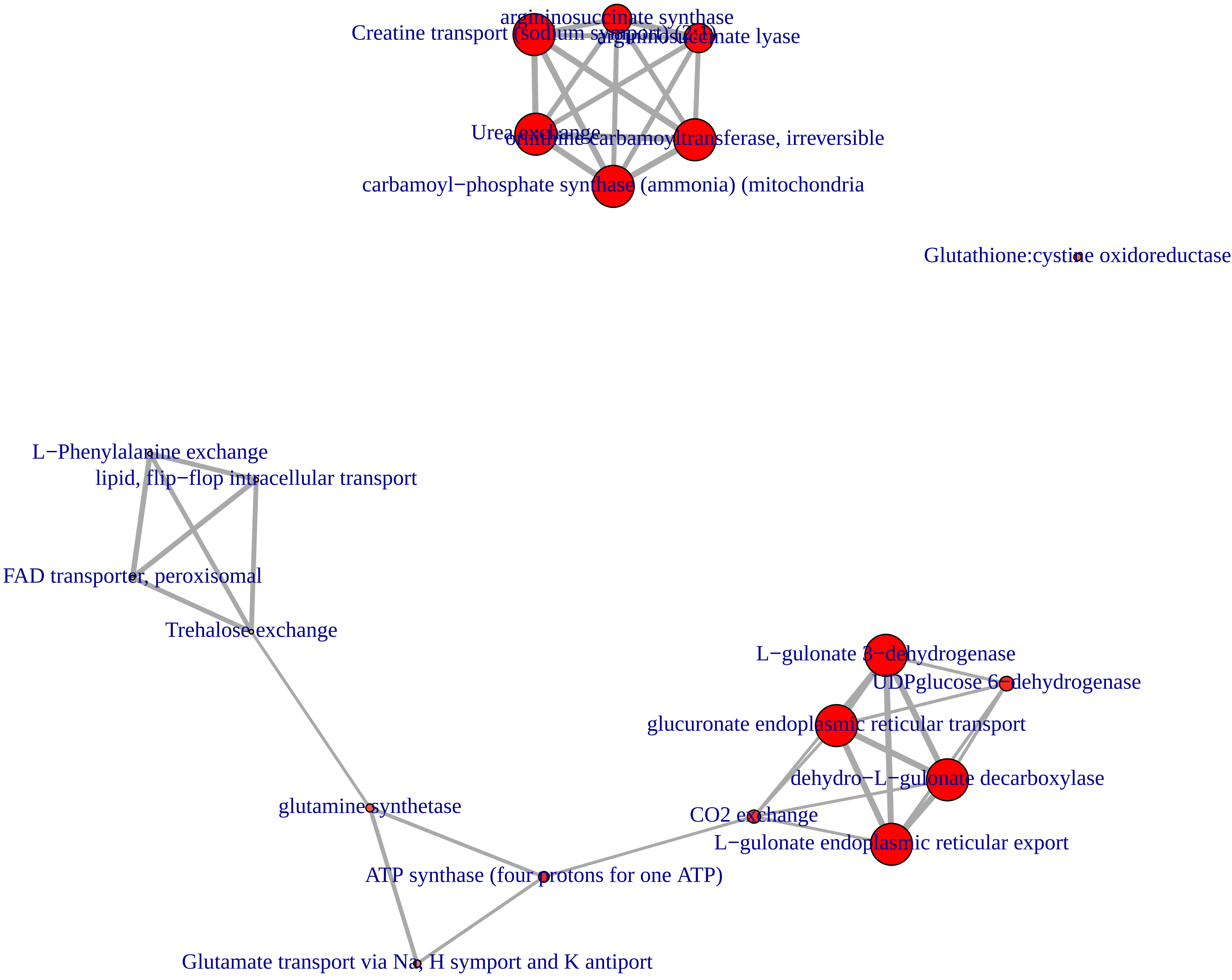
argininosuccinate synthase
Creatine transport (sodium symport) (2:1)
argininosuccinate lyase
Urea exchange
ornithine carbamoyltransferase, irreversible
carbamoyl−phosphate synthase (ammonia) (mitochondria
Glutathione:cystine oxidoreductase
L−Phenylalanine exchange
lipid, flip−flop intracellular transport
FAD transporter, peroxisomal
Trehalose exchange
L−gulonate 3−dehydrogenase
UDPglucose 6−dehydrogenase
glucuronate endoplasmic reticular transport
dehydro−L−gulonate decarboxylase
glutamine synthetase
CO2 exchange
L−gulonate endoplasmic reticular export
ATP synthase (four protons for one ATP)
Glutamate transport via Na, H symport and K antiport
